# Distal Femoral Replacement After Distal Femur Periprosthetic Fracture: A Case Report

**DOI:** 10.7759/cureus.85188

**Published:** 2025-06-01

**Authors:** Rayan Jamal, Hatem A Shaqroon, Mohamad A Ali, Sultan H Farsi

**Affiliations:** 1 Orthopedic Surgery, Prince Mohammed Bin Abdulaziz Hospital, Ministry of the National Guard - Health Affairs, Madinah, SAU; 2 Orthopedic Surgery, King Fahad Hospital, Ministry of Health, Madinah, SAU; 3 General Practice, Saudi German Hospital, Madinah, SAU; 4 Orthopedics, King Salman Armed Forces Hospital, Tabuk, SAU

**Keywords:** distal femoral arthroplasty, knee osteoarthritis, osteoporosis, periprosthetic femur fracture, revision total knee arthroplasty

## Abstract

Distal femoral periprosthetic fractures present a complex challenge, particularly in elderly patients with comorbidities and poor bone quality. We report a case of a 62-year-old Saudi female with morbid obesity and a history of bilateral total knee arthroplasty (TKA) who sustained a distal femoral fracture after minor trauma. Due to limited bone stock and fracture proximity to the prosthesis, internal fixation was ruled out. The patient underwent successful revision TKA with distal femoral replacement (DFR). Postoperative recovery was good, and she was discharged on day 4 with a knee range of motion of 0-90°. This case underscores the viability of DFR as a limb-salvaging option, offering immediate weight-bearing and early mobilization.

## Introduction

With increasing life expectancy and higher functional expectations among the elderly, the number of total knee arthroplasty (TKA) procedures is projected to double within the next decade [1]. As more elderly patients undergo arthroplasty, often with prostheses implanted into the osteoporotic bone, the incidence of periprosthetic fractures is also rising. Managing these fractures is challenging, as most cases require surgical intervention through internal fixation or prosthesis revision. However, fixation of periprosthetic fractures carries a considerable risk of postoperative complications; approximately 10-33% of patients may require additional surgeries due to fixation failure, infection, or a new periprosthetic fracture [2]. If these subsequent interventions fail, patients may ultimately face limb amputation [3]. Nevertheless, endoprosthetic reconstruction (EPR) is increasingly recognized as a more favorable alternative to amputation in such complex cases [2].

Poor bone quality due to osteoporosis and the presence of a femoral component that hinders secure fixation make the management of these fractures particularly challenging [4]. Internal fixation, either with a retrograde intramedullary nail or with plate fixation, remains the primary treatment approach. Revision TKA using a stemmed prosthesis or distal femoral replacement (DFR) is typically reserved for cases involving significant bone loss or a loosened implant [5-8].

Clinical management of supracondylar fractures of the distal femur (type III) has often been associated with poor outcomes, including fixation failure and fracture malunion, leading to ongoing debate regarding the optimal fixation strategy [9]. The use of EPR allows for early mobilization, enabling patients to bear weight immediately following surgery [10].

The clinical outcomes of revision TKA are highly variable, and there remains significant debate over the best approach to managing periprosthetic fractures. Identified risk factors for fixation failure include poor bone quality, malpositioned TKA implants, and fractures located very distally in the femur. Revision arthroplasty offers key advantages in these cases, such as promoting early mobilization and enhancing knee range of motion (ROM) [11]. DFR has been utilized to address these complex fractures, with small case series reporting excellent outcomes and a relatively low incidence of complications [7,12-13].

## Case presentation

A 62-year-old female patient with a medical history of hypertension, hypothyroidism, and morbid obesity (BMI= 40.3 kg/m^2^, class III) presented to the emergency department after a minor trauma sustained nine days earlier. She had undergone bilateral TKA 10 years prior and had been ambulating independently without pain or assistive devices until the injury.

**Figure 1:**
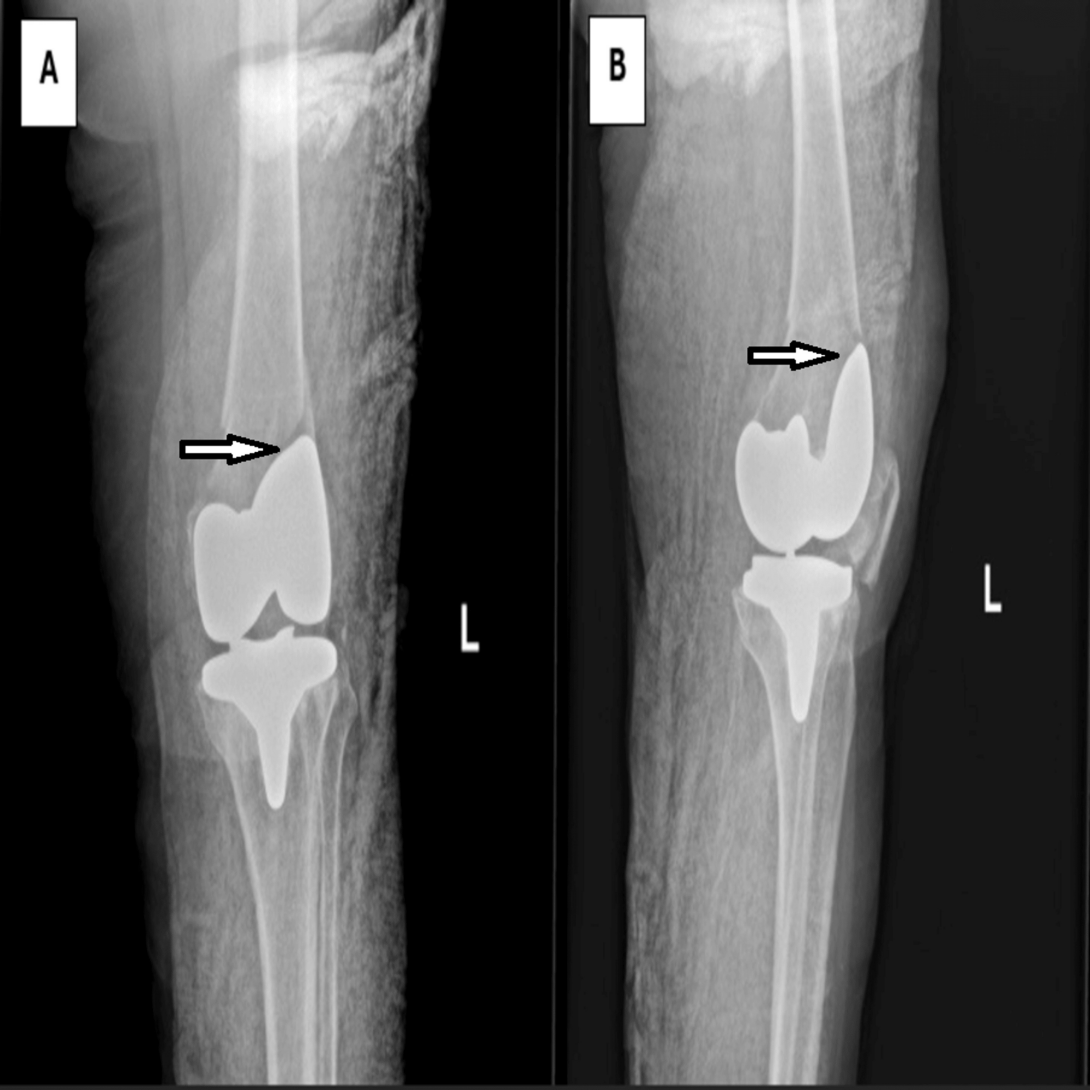
A. Anteroposterior view of the left knee radiograph showing distal femur periprosthetic fracture with unstable component. B. Lateral view of the left knee radiograph showing distal femur periprosthetic fracture with unstable component.

**Figure 2:**
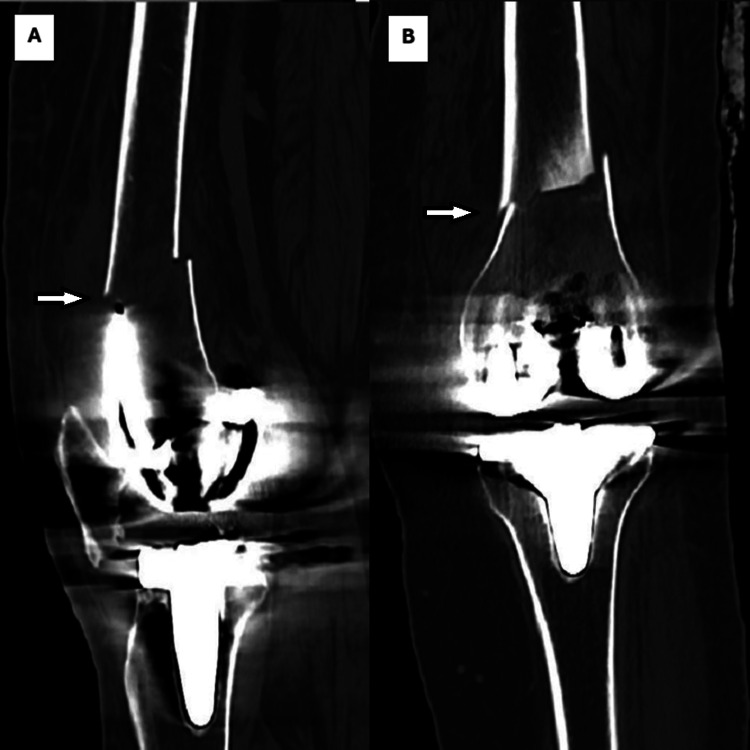
A. Sagittal view of the left knee CT showing distal femur periprosthetic fracture with unstable component. B. Coronal view of the left knee CT showing distal femur periprosthetic fracture with unstable component.

Clinical evaluation revealed the patient was unable to bear weight on the left lower limb, with severe knee pain, significant limitation in ROM, and moderate swelling. Distal neurovascular status was intact. Radiographs showed a distal femoral fracture adjacent to the femoral component of the left TKA (Figure 1). A CT scan confirmed that the distal fragment was short and that the fracture line extended to the anterior cortex, abutting the femoral component (Figure 2).

Due to the fracture pattern and limited bone stock, internal fixation was deemed high-risk for failure and would likely require prolonged immobilization. A revision left TKA with a DFR was planned. Surgery was performed under combined spinal and epidural anesthesia. The patient was positioned supine, and a midline anterior incision through the previous scar was made. A medial parapatellar arthrotomy was performed to access the joint. Intraoperative findings showed the fracture located 5 mm proximal to the joint line and the fracture reaching the femur component, which was found to be loose and unstable with poor bone quality and inadequate bone stock. Partial synovectomy was performed. The tibial component was loose and removed manually. The distal femur fragment was dissected subperiosteally on both medial and lateral aspects.

Sequential intramedullary reaming was performed up to 18 mm for the femur and 14 mm for the tibia. A fully coated, cementless femoral stem (18 mm × 150 mm) with a 25-mm modular extension and standard 75-mm body was implanted. For the tibia, a size 4 tibial baseplate with a fully coated cementless stem (14 mm × 150 mm) and a standard polyethylene insert were used. Intraoperative assessment showed full extension, satisfactory ROM, good component rotation, and appropriate patellar tracking. Layered wound closure was performed over two drains.

The patient tolerated the procedure well and was transferred to recovery in stable condition. Ankle pump exercises were initiated on the same day. Prophylactic antibiotics were given for 48 hours. Full weight-bearing was allowed from postoperative day 1. Drain outputs were as follows: the deep drain (intra-articular) produced 250 mL in the first 24 hours and 50 mL the next day; it was removed on day 2. The superficial drain yielded 200 mL initially and 10 mL the next day; it was also removed on day 2.

**Figure 3:**
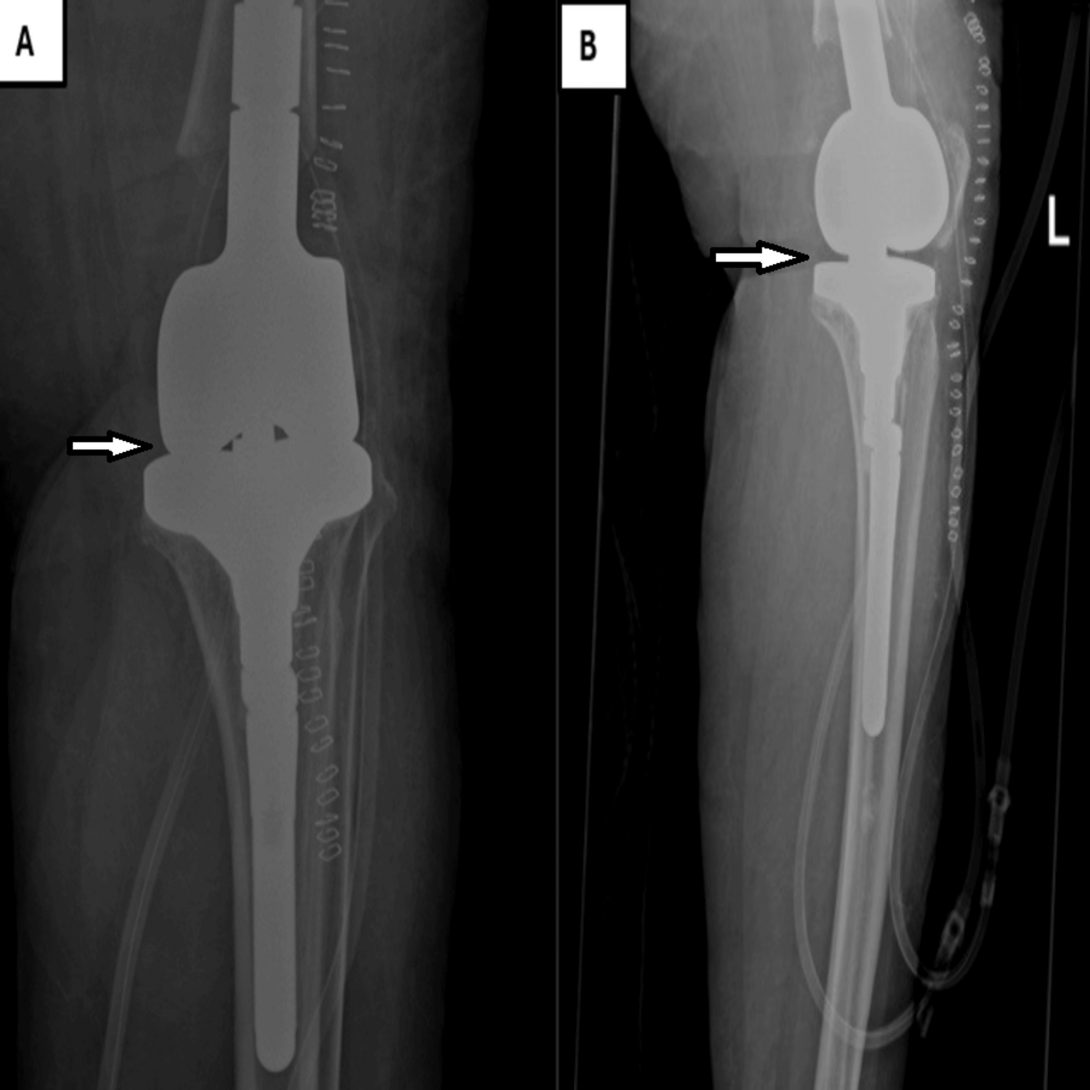
Left knee radiograph post-TKA revision with distal femur replacement and tibial component revision with long stem showing adequate restoration of joint space and well-aligned and fixed components (A: anteroposterior view; B: lateral view). TKA, total knee arthroplasty

**Figure 4:**
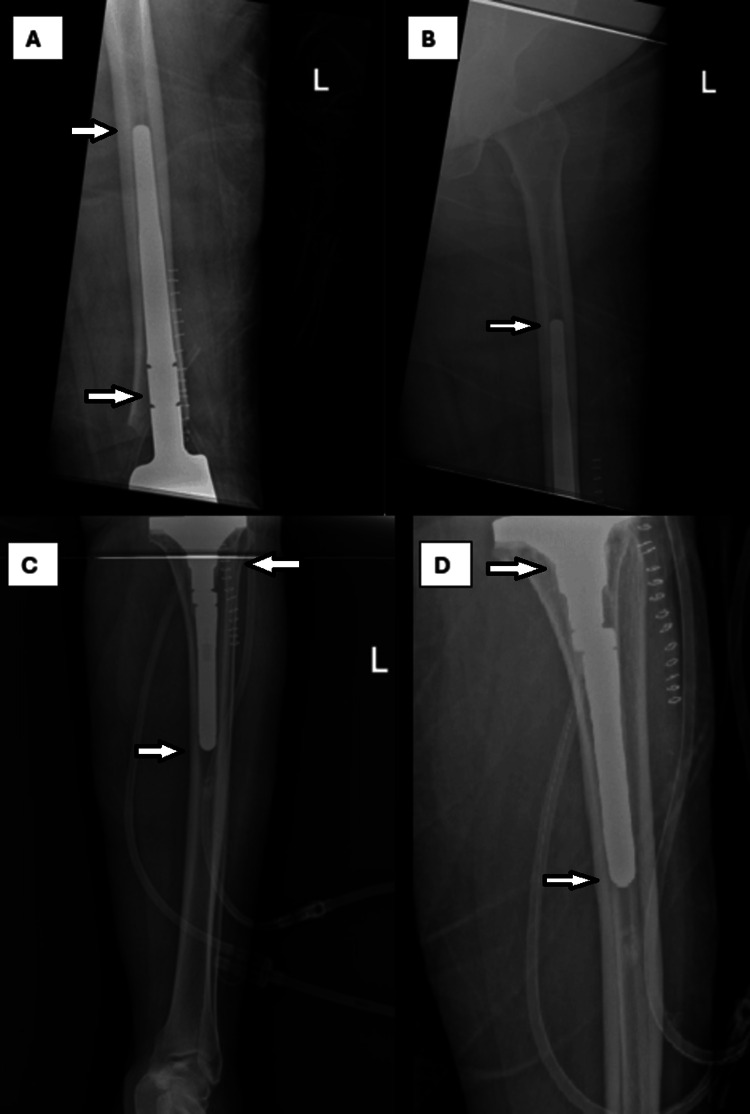
Left femur radiograph post-TKA revision with distal femur replacement showing the femoral prothesis in adequate position with the implant tip reaching the upper half of the femur and no penetration to the cortex (A: anteroposterior view of the lower half; B: anteroposterior view of the upper half). Left tibia radiograph post-TKA revision showing tibial component with long stem in adequate position with well aligned and fixed components and no penetration of the tibial cortex (C: anteroposterior view; D: lateral view). TKA, total knee arthroplasty

Post-operative X-ray was performed the next day, which showed adequate and satisfactory results for both distal femur prosthesis and tibial component (Figures 3, 4).

The postoperative course was uneventful. The patient was discharged on postoperative day 4 in stable condition with an ROM of 0-90 degrees. She was instructed to continue outpatient physiotherapy and to attend follow-up visits at two weeks (for suture removal and ROM assessment), six weeks, and three months postoperatively.

## Discussion

Periprosthetic fractures after TKA represent a complex challenge, especially in the presence of poor bone quality, implant loosening, or malalignment. These injuries are predominantly seen in elderly patients with multiple comorbidities, who are typically incapable of withstanding extended immobility or weight-bearing limitations. Compromised bone healing further exacerbates the risk of treatment failure [14]. Therefore, achieving a stable limb is critical, as early mobilization can significantly reduce medical complications [15].

There is no universally accepted protocol for managing supracondylar periprosthetic femoral fractures (PPFs). Still, open or closed reduction with internal fixation is usually considered the initial treatment of choice [16]. This approach, however, becomes inappropriate when the femoral component is unstable or the fracture is extensively comminuted, especially in the presence of deficient bone stock. In such situations, DFR emerges as a feasible surgical solution, particularly for patients with loose components and compromised distal femoral bone [17].

Mortazavi et al. [18] highlighted that PPF patients following TKA are generally older and medically complex. Surgical exploration often reveals extensive bone loss and ligamentous insufficiency, necessitating hinged prostheses. DFR implants offer the ability to restore bone integrity, realign the limb, and reestablish joint stability [18]. Postoperative outcomes showed consistent pain relief, improved joint function, and regained mobility, with patients returning to their pre-injury activity levels [18].

Treatment strategies for supracondylar femoral fractures include internal fixation and revision arthroplasty. Retrograde intramedullary nailing is typically indicated for high supracondylar fractures, particularly Su type I and selected type II fractures, assuming the femoral prosthesis allows nail passage. In contrast, locking plate fixation is commonly used for Su type II and some type III fractures [19]. For elderly, low-demand patients with compromised bone stock, especially in Su type III fractures, DFR is advised [20,21]. DFR offers several key benefits such as early mobilization, accelerated recovery, and immediate weight-bearing postoperatively [18]. Unlike fixation techniques, DFR bypasses the need for biological bone healing, which is frequently impaired in geriatric populations. Additionally, DFR demonstrates a reduced incidence of medium-term complications, including aseptic loosening [21, 22].

In the presented case of a 62-year-old female with multiple comorbidities, the patient sustained a short distal femoral fragment with the fracture extending to the anterior cortex, approaching the femoral component. Given these features and the patient’s overall condition, internal fixation was deemed suboptimal due to its high failure risk and the need for prolonged immobilization. Revision TKA with DFR was chosen to restore limb alignment, achieve immediate stability, and enable early mobilization. Intraoperatively, a loose tibial component was identified, justifying a comprehensive revision. Fully coated, cementless stems were employed for both components to achieve durable fixation.

In our case, the surgical findings confirmed restored mechanical alignment, optimal implant positioning, satisfactory patellar tracking, and no intraoperative complications. The patient demonstrated an acceptable ROM. Postoperatively, she initiated full weight-bearing on the first day, highlighting one of the main advantages of EPR in high-risk patients, where early mobilization is crucial to reduce morbidity. The patient experienced an uncomplicated early recovery.

Historically, locking plates and retrograde intramedullary nails for distal femoral periprosthetic fractures have shown high failure rates and required prolonged immobilization for union. Campbell et al. in a multicenter study including 55 patients reported a 24% complication rate and 18% nonunion rate with precontoured locking plates [23]. Hoffmann et al. found a 30.6% nonunion rate in 36 locking plate cases across two centers [24]. Shin et al. reviewed outcomes of locking compression plating versus retrograde nailing, noting limitations in both techniques [25]. Another review of 41 studies reported a 35% complication rate for locking plates and 53% for retrograde nails in Rorabeck type II fractures [26]. Locking plates, though commonly used, carry high risks of complications and nonunion.

Conversely, a retrospective study reported that DFRs used for comminuted periprosthetic distal femur fractures resulted in favorable clinical outcomes and a low complication rate in this complex patient group. Successful outcomes with EPR are highly dependent on thorough preoperative planning and precise surgical execution [27].

Patients at high risk for nonunion or with compromised bone quality are prime candidates for primary treatment with DFR. Other key indications include very distal periprosthetic femur fractures, implant loosening, substantial bone loss, inability to adhere to weight-bearing restrictions, and patients with serious comorbidities (e.g., obesity, hypertension, hypothyroidism) who are suited for a single definitive surgery. In one study, open reduction and internal fixation failed in 9.2% of cases, with leading failure causes being nonunion (53.8%), infection (30.8%), plate loosening (7.7%), and refracture (7.7%) [28].

## Conclusions

DFR is a viable and effective option for managing periprosthetic distal femur fractures with severe bone loss, especially in patients with multiple comorbidities. It minimizes the risk of fixation failure, allows early mobilization, and leads to satisfactory functional outcomes when performed with proper surgical technique. The key advantages include reliable structural support, reduced complications compared to internal fixation in poor bone stock, and faster return to mobility. Despite its benefits, the procedure requires meticulous preoperative planning, including detailed imaging, implant selection, and anticipation of soft tissue challenges. Precise surgical execution remains critical to achieving favorable outcomes in these complex and high-risk cases.

## References

[1] Kurtz SM, Ong KL, Lau E, Bozic KJ (2014). Impact of the economic downturn on total joint replacement demand in the United States: updated projections to 2021. J Bone Joint Surg Am.

[2] Agrawal AC, S L, Sakale H, Narayan Dash R, Chauhan S (2024). Total femoral replacement in periprosthetic femur fracture: a case report. Cureus.

[3] Pasquina PF, Miller M, Carvalho AJ, Corcoran M, Vandersea J, Johnson E, Chen YT (2014). Special considerations for multiple limb amputatio. Curr Phys Med Rehabil Rep.

[4] Ricci WM, Borrelli J Jr (2007). Operative management of periprosthetic femur fractures in the elderly using biological fracture reduction and fixation techniques. Injury.

[5] Bengoa F, Neufeld ME, Howard LC, Masri BA (2023). Periprosthetic fractures after a total knee arthroplasty. J Am Acad Orthop Surg.

[6] Srinivasan K, Macdonald DA, Tzioupis CC, Giannoudis PV (2005). Role of long stem revision knee prosthesis in periprosthetic and complex distal femoral fractures: a review of eight patients. Injury.

[7] Saidi K, Ben-Lulu O, Tsuji M, Safir O, Gross AE, Backstein D (2014). Supracondylar periprosthetic fractures of the knee in the elderly patients: a comparison of treatment using allograft-implant composites, standard revision components, distal femoral replacement prosthesis. J Arthroplasty.

[8] Jassim SS, McNamara I, Hopgood P (2014). Distal femoral replacement in periprosthetic fracture around total knee arthroplasty. Injury.

[9] Kancherla VK, Nwachuku CO (2014). The treatment of periprosthetic femur fractures after total knee arthroplasty. Orthop Clin North Am.

[10] Höll S, Schlomberg A, Gosheger G, Dieckmann R, Streitbuerger A, Schulz D, Hardes J (2012). Distal femur and proximal tibia replacement with megaprosthesis in revision knee arthroplasty: a limb-saving procedure. Knee Surg Sports Traumatol Arthrosc.

[11] Kuzyk PR, Watts E, Backstein D (2017). Revision total knee arthroplasty for the management of periprosthetic fractures. J Am Acad Orthop Surg.

[12] Berend KR, Lombardi AV Jr (2009). Distal femoral replacement in nontumor cases with severe bone loss and instability. Clin Orthop Relat Res.

[13] Keenan J, Chakrabarty G, Newman JH (2000). Treatment of supracondylar femoral fracture above total knee replacement by custom made hinged prosthesis. Knee.

[14] Gruber R, Koch H, Doll BA, Tegtmeier F, Einhorn TA, Hollinger JO (2006). Fracture healing in the elderly patient. Exp Gerontol.

[15] Zuckerman JD, Skovron ML, Koval KJ, Aharonoff G, Frankel VH (1995). Postoperative complications and mortality associated with operative delay in older patients who have a fracture of the hip. J Bone Joint Surg Am.

[16] Al-Jabri T, Ridha M, McCulloch RA, Jayadev C, Kayani B, Giannoudis PV (2023). Periprosthetic distal femur fractures around total knee replacements: a comprehensive review. Injury.

[17] Sobol KR, Fram BR, Strony JT, Brown SA (2022). Survivorship, complications, and outcomes following distal femoral arthroplasty for non-neoplastic indications. Bone Jt Open.

[18] Mortazavi SM, Kurd MF, Bender B, Post Z, Parvizi J, Purtill JJ (2010). Distal femoral arthroplasty for the treatment of periprosthetic fractures after total knee arthroplasty. J Arthroplasty.

[19] Su ET, DeWal H, Di Cesare PE (2004). Periprosthetic femoral fractures above total knee replacements. J Am Acad Orthop Surg.

[20] Kim KI, Egol KA, Hozack WJ, Parvizi J (2006). Periprosthetic fractures after total knee arthroplasties. Clin Orthop Relat Res.

[21] Johnston AT, Tsiridis E, Eyres KS, Toms AD (2012). Periprosthetic fractures in the distal femur following total knee replacement: a review and guide to management. Knee.

[22] Harrison RJ Jr, Thacker MM, Pitcher JD, Temple HT, Scully SP (2006). Distal femur replacement is useful in complex total knee arthroplasty revisions. Clin Orthop Relat Res.

[23] Campbell ST, Lim PK, Kantor AH (2020). Complication rates after lateral plate fixation of periprosthetic distal femur fractures: a multicenter study. Injury.

[24] Hoffmann MF, Jones CB, Sietsema DL, Koenig SJ, Tornetta P 3rd (2012). Outcome of periprosthetic distal femoral fractures following knee arthroplasty. Injury.

[25] Shin YS, Kim HJ, Lee DH (2017). Similar outcomes of locking compression plating and retrograde intramedullary nailing for periprosthetic supracondylar femoral fractures following total knee arthroplasty: a meta-analysis. Knee Surg Sports Traumatol Arthrosc.

[26] Ebraheim NA, Kelley LH, Liu X, Thomas IS, Steiner RB, Liu J (2015). Periprosthetic distal femur fracture after total knee arthroplasty: a systematic review. Orthop Surg.

[27] Matar HE, Bloch BV, James PJ (2021). Distal femoral replacements for acute comminuted periprosthetic knee fractures: satisfactory clinical outcomes at medium-term follow-up. Arthroplast Today.

[28] Chen AF, Choi LE, Colman MW, Goodman MA, Crossett LS, Tarkin IS, McGough RL (2013). Primary versus secondary distal femoral arthroplasty for treatment of total knee arthroplasty periprosthetic femur fractures. J Arthroplasty.

